# The system of radiological protection and the UN sustainable development goals

**DOI:** 10.1007/s00411-024-01089-w

**Published:** 2024-09-10

**Authors:** W. Rühm, K. Applegate, F Bochud, D Laurier, T. Schneider, S. Bouffler, K. Cho, C. Clement, O. German, G. Hirth, M. Kai, S. Liu, A. Mayall, S. Romanov, A. Wojcik

**Affiliations:** 1 Federal Office for Radiation Protection, Ingolstädter Landstraße 1, Neuherberg, D-85764 Germany; 2 University of Kentucky College Medicine, 800 Rose Street MN 150, Lexington, Kentucky 40506 USA; 3 Institute of Radiation Physics, Lausanne University Hospital and University of Lausanne, Rue du Grand-Pré 1, Lausanne, CH-1007 Switzerland; 4 Institut de radioprotection et de Sûreté Nucléaire, BP 17 - 92262 Fontenay-aux-Roses Cedex, 31 avenue de la Division Leclerc, Fontenay-aux-Roses, Île-de-France 92260 France; 5 Nuclear Protection Evaluation Centre, 28, rue de la Redoute, Fontenay aux Roses, F-92260 France; 6 UK Health Security Agency, Radiation Protection Sciences Division, Didcot, Oxon, OX11 0RQ UK; 7 Korea Institute of Nuclear Safety, PO Box 114, Yuseong, Daejeon, 34142 Korea; 8 International Commission on Radiological Protection, 350 Albert Street, Ottawa, Ontario K1R 1A4 Canada; 9 Australian Radiation Protection and Nuclear Safety Agency, 619 Lower Plenty Road, Yallambie, VIC 3085 Australia; 10 Nippon Bunri University, 1727 Ichigi, Ōita, 870-0397 Japan; 11 China Institute of Atomic Energy, PO Box 275 (1), Beijing, CN-102413 People’s Republic of China; 12 Environment Agency, Ghyll Mount, Gillan Way, Penrith, Cumbria CA11 9BP UK; 13 Southern Urals Biophysics Institute, Ozyorsk, Chelyabinsk Region Russian Federation; 14 Centre for Radiation Protection Research, Stockholm University, Svante Arrheniusväg 20C, Stockholm, 106 91 Sweden; 15 Institute of Biology, Jan Kochanoski University, Kielce, 25-406 Poland

**Keywords:** UN Sustainable Development Goals, SDGs, System of Radiological Protection, International Commission on Radiological Protection, ICRP

## Abstract

In 2015 the United Nations issued 17 Sustainable Development Goals (SDGs) addressing a wide range of global social, economic, and environmental challenges. The main goal of this paper is to provide an understanding of how the current System of Radiological Protection relates to these SDGs. In the first part it is proposed that the current System of Radiological Protection is implicitly linked to sustainable development. This is substantiated by analysing the features of the current System as set out by the International Commission on Radiological Protection (ICRP) in its publications. In the second part it is proposed that sustainability should be considered and more explicitly addressed in the next ICRP general recommendations, as part of the currently ongoing review and revision of the current System. A few examples are given of how this could be realised, and it is proposed that this issue should be discussed and developed together with the international community interested in radiological protection.

## Introduction

In 2015 the United Nations issued the Sustainable Development Goals (SDGs) as part of the 2030 Agenda for Sustainable Development. The Agenda aims to address a wide range of social, economic, and environmental challenges. It provides “*a shared blueprint for peace and prosperity for people and the planet*,* now and into the future. At its heart are the 17 Sustainable Development Goals (SDGs)*,* which are an urgent call for action by all countries - developed and developing - in a global partnership. They recognize that ending poverty and other deprivations must go hand-in-hand with strategies that improve health and education*,* reduce inequality*,* and spur economic growth – all while tackling climate change and working to preserve our oceans and forests*.” A detailed description of these SDGs including 169 associated targets, can be found in (UN, 2015). Each goal has specific targets and indicators to measure progress. As these goals are often interrelated, actions taken to achieve one goal may have positive or negative impacts on other goals. Applying them therefore requires holistic and systematic thinking.

The 2030 Agenda of the United Nations encourages cooperation and partnerships among governments, industry, civil society, and individuals to work together to achieve these global goals by 2030. As radiological protection is a cross-cutting issue that involves all these entities, the International Commission on Radiological Protection (ICRP) decided to analyse the interrelationship between the SDGs and the current System of Radiological Protection (the “System”) set out in ICRP Publication 103 (ICRP 2007) and publish its vision on the subject. Any future incorporation of the SDGs into the System is all the more timely as ICRP has recently initiated the process of reviewing and revising the System (Clement et al. 2021).

This paper is not intended to provide an in-depth, historical review of the links between radiological protection in general and sustainable development. Rather, the main goal of this paper is to describe how the current System of Radiological Protection relates to the SDGs. Furthermore, looking into the future, some important questions are: can SDGs be considered as drivers for the next general recommendations following ICRP Publication 103 (ICRP 2007)? If yes, how can they be integrated? To achieve this goal and find answers to these questions, we first illustrate ICRP’s commitments to the SDGs in its current recommendations and its most recent publications, as well as in its governance. In this context one should keep in mind the primary mission of ICRP, which is to contribute to an appropriate level of protection for people and the environment against the detrimental effects of ionising radiation exposure without unduly limiting the benefits associated with the use of radiation. We then outline possible ways to strengthen the alignment of the System with sustainable development. The paper is also meant to stimulate discussion with other ICRP’s stakeholders and other international organisations interested in radiological protection on the way the System already addresses and could in the future further address the concept of sustainability.

## The current system of radiological protection and sustainability

## Setting the scene

As is shown further below, the current System implicitly includes some elements of the sustainable development concept. For example, the need to balance the social and economic benefits of radiation use with its potential harm is recognised in the primary aim of the current System. In addition, aspects such as long-term impact (related to intergenerational equity) are considered in areas such as the disposal of radioactive waste. However, with the growing and urgent need to address global challenges it is important to consider further how radiological protection in general and the System in particular contribute to sustainable development and how this might be enhanced; and whether the primary aim of radiological protection should be amended to make its contribution to sustainable development explicit (Mayall 2022).

A recent series of publications have brought forward core and procedural ethical values used in the System. In ICRP Publication 138 it is stated that “*Ethics cannot provide conclusive solutions*,* but can help to facilitate discussions among those seeking to promote the well-being of individuals*,* the sustainable development of society*,* and the protection of the environment*” (ICRP 2018a, para7).

Publication 138 provides an ethical framework for radiological protection, implicitly echoing many of the SDGs. The four core ethical values are summarised hereafter:

- Beneficence/non maleficence: promoting or doing good, and avoiding doing harm;
- Prudence: making informed and carefully considered choices without full knowledge of the scope and consequences of an action;
- Justice: fairness in the distribution of advantages and disadvantages;
- Dignity: the unconditional respect that every person deserves, irrespective of personal attributes or circumstances.

Publication 138 also points out the importance of “*accountability of the present generation to future generations*” (ICRP 2018a; Para. 68).

As mentioned above, the SDGs as set out by the UN are not independent from each other, and reaching some of the goals may facilitate or attenuate the prospect of attaining others. Use of ionising radiation and development of radiological protection guidelines face a similar problem: today, the use of ionising radiation contributes to the sustainability of our modern way of life, but may at the same time compromise the sustainable use of resources. One example is the contribution of radiation to both diagnostic and therapeutic medicine (UNSCEAR 2022) supporting SDG 3 (Good Health and Well-Being). But the other side of the coin cannot be ignored: Access to health care is far from being equitable, as are other aspects of our society such as access to food, clothing, shelter, energy, education, jobs, safety from violence, etc. (UNSCEAR 2022). The World Health Organisation (WHO) Universal Health Coverage indicated that *“the proportion of the population not covered by essential health services decreased by about 15% between 2000 and 2021*,* with minimal progress made after 2015. This indicates that in 2021*,* about 4.5 billion people were not fully covered by essential health services***”.** Moreover, “*about 2 billion people are facing financial hardship including 1 billion experiencing catastrophic out-of-pocket health spending (SDG indicator 3.8.2) or 344 million people going deeper into extreme poverty due to health costs***”** (WHO 2023). On the other hand, overdiagnosis and overtreatment are commonplace demonstrating wastefulness of resources (Kühlein et al. 2023). What’s more, health care (in which various types of radiation play a significant role) causes global environmental impacts with regard to emission of greenhouse gases, particulate matter, nitrogen oxides (NO_x_), sulphur dioxide (SO_2_), etc., ranging between 1% and 5% of total global impacts, and are more than 5% for some national impacts (Lenzen et al. 2020; Picano et al. 2023). Picano et al. estimate the contribution of healthcare to total national carbon footprint (4% in the UK, 10% in USA) and medical imaging (approximately 10% of healthcare CO_2_ emissions in the US) to overall carbon emissions on a planetary scale.

As another example of the double-edged nature of SDGs in relation to ionising radiation, nuclear energy emits less greenhouse gas than fossil fuel energy generation (IEA 2019). However, nuclear accidents are possible (albeit with low probability). This may compromise the well-being of locally affected people by direct health effects, psychological stress or the potential economic blight resulting from such an accident or its countermeasures, extending sometimes to regional or even global effects. Furthermore, most countries have not achieved a consensus for a long-term solution for high-activity radioactive waste (see also Böse et al. 2024; Wimmer et al. 2024). Generally, the various forms of energy production available or under development such as the use of renewable energy sources or fusion have advantages and disadvantages (see, for example, https://ourworldindata.org/safest-sources-of-energy for a discussion regarding human health and climate change). Radiological protection might support the use of many forms given the fact that many technologies of energy production can result in exposure to ionising radiation (UNSCEAR 2017). While ICRP is not in a position to make or propose decisions on the most appropriate energy generation technologies, its System of Radiological Protection will support the safe use of radiation sources in any context and recommend strategies to provide appropriate protection from the detrimental effects of radiation.

### Publications of ICRP in relation to SDGs

Overall, the goal of the System can be paraphrased as promoting ethics-based, science-based, and experience-based approaches to ensuring the health and safety of individuals and the environment in the presence of ionising radiation. The current general recommendations (the “Recommendations”) set out in ICRP Publication 103 (ICRP 2007) do not explicitly mention sustainability, but a closer look reveals that many SDGs are implicitly present.

In the remainder of this section, we show where the SDGs are implicitly present in the current Recommendations and, by way of example, how some more recent ICRP publications support several SDGs more or less explicitly. Further examples are shown in Table 1. This is important from the perspective of the possible further integration of the SDGs into the new Recommendations (see also section “Towards the next general recommendations”).

**Table 1:** List of the SDGs with examples of use or existence of ionising radiation which require recommendations on radiological protection

Sustainable Development Goals (SDGs)	Subgoals (examples)	Examples of use or existence of ionising radiation which require recommendations on radiological protection
1. End poverty in all its forms everywhere	1.5 Build the resilience of the poor and those in vulnerable situations and reduce their exposure and vulnerability to climate-related extreme events and other economic, social and environmental shocks and disasters	Resilience of people in vulnerable situations after nuclear emergencies: -ICRP Publication 146 “Radiological protection of people and the environment in the event of a large nuclear accident” (ICRP 2020d): Recommends optimal protection of people and the environment in a large nuclear accident, drawing on experience of the Chernobyl and Fukushima accidents. -ICRP Publication 109 “Application of the Commission’s Recommendations for the Protection of People in Emergency Exposure Situations” (ICRP 2009a): Provides guidance for optimal preparedness for, and response to, all radiation emergency exposure situations that require urgent action in order to avoid or reduce undesirable consequences. - ICRP Publication 111 “Application of the Commission’s Recommendations to the Protection of People Living in Long-term Contaminated Areas after a Nuclear Accident or a Radiation Emergency” (ICRP 2009b): Provides guidance for optimal protection of people living in long-term contaminated areas resulting from either a nuclear accident or a radiation emergency.
2. End hunger, achieve food security and improved nutrition and promote sustainable agriculture	2.4 Ensure sustainable food production systems and implement resilient agricultural practices that increase productivity and production, that help maintain ecosystems, that strengthen capacity for adaptation to climate change, extreme weather, drought, flooding and other disasters, and that progressively improve land and soil quality	Remediation of contaminated arable land and restoration of agricultural activities following a nuclear accident or for the management of legacy sites: - Proceedings of the International Conference on Recovery after Nuclear Accidents: Radiological Protection Lessons from Fukushima and Beyond (ICRP 2021e): Include recommendations on remediation of contaminated arable land and restoration of agricultural activities following a nuclear accident or for the management of legacy sites - ICRP Publication 124 “Protection of the environment under different exposure situations” (ICRP 2014a): Describes the framework for protection of the environment and explains how the Commission’s recommendations can be implemented to satisfy different forms of environmental protection objectives.
3. Ensure healthy lives and promote well-being for all at all ages	3.4 Reduce by one third premature mortality from non-communicable diseases through prevention and treatment and promote mental health and well-being 3.9. Reduce the number of deaths and illness from hazardous chemicals and air, water and soil pollution and contamination	Safe use of ionising radiation in medical imaging and radiotherapy: - ICRP Publication 135 “Diagnostic reference levels in medical imaging” (ICRP 2017a): Recommends quantities for use as diagnostic reference level (DRLs) for various imaging modalities and provides information on the use of DRLs for interventional procedures and in paediatric imaging. - ICRP Publication 140 “Radiological protection in therapy with radiopharmaceuticals” (ICRP 2019a): Provides an overview of therapeutic procedures and a framework for calculating radiation doses for various treatment approaches to ensure optimal treatment outcome.
4. Ensure inclusive and equitable quality education and promote lifelong learning opportunities for all	4.3 Ensure equal access for all women and men to affordable and quality technical, vocational and tertiary education, including university	Support of Education & training in radiological protection: - ICRP Mentorship Programme (https://www.icrp.org/page.asp?id=465): To promote knowledge in the field of radiological protection among young people worldwide. - ICRP Vancouver Call for Action to strengthen expertise in radiological protection worldwide (Rühm et al. 2023a) - ICRP Publication 154 “Optimisation of Radiological Protection in Digital Radiology Techniques for Medical Imaging”, in press (to include education/training, teamwork, lifelong learning, assessment of quality assurance and improvement) (ICRP 2024b) and ICRP Publication “Practical Aspects in Optimisation of Radiological Protection in Digital Radiography, Fluoroscopy, and CT”, in press (ICRP 2024g) - ICRPaedia educational materials (http://icrpaedia.org/Main_Page) - Web-based open events organised by ICRP and free publications and training materials available at the ICRP website: To offer open and free-of charge education in the field of radiological protection among people worldwide.
6. Ensure availability and sustainable management of water and sanitation for all	6.3 Improve water quality by reducing pollution, eliminating dumping and minimising release of hazardous chemicals and materials, halving the proportion of untreated wastewater and substantially increasing recycling and safe reuse globally.	Radiological protection of the environment including water resources: - ICRP Publication 124 “Protection of the environment under different exposure situations” (ICRP 2014a): Recommends how different forms of environmental protection objectives can be reached, which may require the use of representative organisms specific to a site, and how these may be compared with the reference values.
8. Promote sustained, inclusive and sustainable economic growth, full and productive employment and decent work for all	8.2 Achieve higher levels of economic productivity through diversification, technological upgrading and innovation, including through a focus on high-value added and labour-intensive sectors	Radiation protection in technological developments: - ICRP Publication 123 “Assessment of Radiation Exposure of Astronauts in Space” (ICRP 2013b): Describes the terms and methods used to assess the radiation exposure of astronauts and provides data for the assessment of organ doses to optimise health protection. - ICRP Publication 132 “Radiological protection from cosmic radiation in aviation” (ICRP 2016a): Describes the basic radiological protection principles that apply to passengers and aircraft crew and the available protective actions. - ICRP Publication 142 “Radiological protection from naturally occurring radioactive material (NORM) in industrial processes” (ICRP 2019c): Provides guidance on radiological protection in industries involving naturally occurring radioactive material (NORM). - ICRP Publication 154 “Optimisation of Radiological Protection in Digital Radiology Techniques for Medical Imaging” (ICRP 2024b): Provides guidance on the adaptation of levels of dose and image quality to clinical tasks, taking advantage of the wide dynamic range offered by digital imaging equipment.
10. Reduce inequality within and among countries	10.2 Empower and promote the social, economic and political inclusion of all, irrespective of age, sex, disability, race, ethnicity, origin, religion or economic or other status	International collaborations and engagement (see also SDGs 4 and 5): - ICRP recommendations to be applied all over the world for the benefit of all. - ICRP Publication 138 “Ethical Foundations of the System of Radiological Protection” (ICRP 2018a): Development of a System of Radiological Protection that is based on fundamental ethical principles.
11. Make cities and human settlements inclusive, safe, resilient and sustainable	11.1 Ensure access for all to adequate, safe and affordable housing and basic services and upgrade slums	Protection against exposure due to radon at home and at work: - ICRP Publication 65 “Protection Against Radon-222 at Home and at Work” (ICRP 1993): Recommends the control of radon and its progeny in both dwellings and workplaces. - ICRP Publication 126 “Radiological Protection against Radon Exposure” (ICRP 2014b): Describes the characteristics of radon exposure, covering sources and transfer mechanisms, the health risks associated with radon, and the challenges of managing radon exposure.
12. Ensure sustainable consumption and production patterns	12.4 Achieve the environmentally sound management of chemicals and all wastes throughout their life cycle, in accordance with agreed international frameworks, and significantly reduce their release to air, water and soil in order to minimise their adverse impacts on human health and the environment	Recommendations on the management of radioactive waste in the environment: - ICRP Publication 122 “Radiological Protection in Geological Disposal of Long-lived Solid Radioactive Waste” (ICRP 2013a): Explains how the ICRP System of Radiological Protection described in Publication 103 can be applied in the context of the geological disposal of long-lived solid radioactive waste. - ICRP Publication 142 “Radiological protection from naturally occurring radioactive material (NORM) in industrial processes” (ICRP 2019c): Provides guidance on radiological protection in industries involving NORM.
14. Conserve and sustainably use the oceans, seas and marine resources for sustainable development	14.2 Sustainably manage and protect marine and coastal ecosystems to avoid significant adverse impacts including by strengthening their resilience, and take action for their restoration in order to achieve healthy and productive oceans	Radiological Protection of the Environment through radiological impact assessment: - ICRP Publication 91 “A Framework for Assessing the Impact of Ionising Radiation on Non-human Species” (ICRP 2003), ICRP Publication 108 “Environmental Protection - the Concept and Use of Reference Animals and Plants” (ICRP 2008), ICRP Publication 124 “Protection of the environment under different exposure situations” (ICRP 2014a): Methodological framework for sea water reference animals (fish, crustaceans) based on scientific evidence on the effects of ionising radiation on non-human populations; Provide guidelines for measuring the impact of radiation on the environment to optimise protection.
15. Protect, restore and promote sustainable use of terrestrial ecosystems, sustainably manage forests, combat desertification, and halt and reverse land degradation and halt biodiversity loss	15.1 Ensure the conservation, restoration and sustainable use of terrestrial and inland freshwater ecosystems and their services, in particular forests, wetlands, mountains and drylands, in line with obligations under international agreements	Radiological Protection of the Environment through radiological impact assessment: - ICRP Publication 91 “A Framework for Assessing the Impact of Ionising Radiation on Non-human Species” (ICRP 2003), ICRP Publication 108 “Environmental Protection - the Concept and Use of Reference Animals and Plants” (ICRP 2008), ICRP Publication 153 “Radiological protection in veterinary practice” (ICRP 2022c): Methodological framework for terrestrial reference plants; Provide guidelines for measuring the impact of radiation on the environment to optimise protection.
16. Promote peaceful and inclusive societies for sustainable development, provide access to justice for all and build effective, accountable and inclusive institutions at all levels	16.7. Ensure responsive, inclusive, participatory and representative decision-making at all levels	Stakeholder’ participation in RP decision making: - ICRP Publication 138 “Ethical foundations of the System of Radiological Protection” (ICRP 2018a): Seen as a procedural value in the System of Radiological Protection - Initiation of the Fukushima dialogue (ICRP 2016d “Proceedings of the International Workshop on the Fukushima dialogue Initiative”) - ICRP Publication 146 “Radiological protection of people and the environment in the event of a large nuclear accident” (ICRP 2020d): Promotion of the co-expertise process with stakeholders in affected areas - Publication 154 “Optimisation of Radiological Protection in Digital Radiology Techniques for Medical Imaging” (ICRP 2024b): Discusses shared decision making and patient well-being
17. Strengthen the means of implementation and revitalize the Global Partnership for Sustainable Development	17.6 Enhance North-South, South-South and triangular regional and international cooperation on and access to science, technology and innovation and enhance knowledge sharing on mutually agreed terms, including through improved coordination among existing mechanisms, in particular at the United Nations level, and through a global technology facilitation mechanism	International relationship and formal relations with Special Liaison Organisations: - Development of the System of Radiological Protection in close contact with various international organisations including UN organisations and professional associations (Rühm et al. 2022, 2023b) - Informal relationships with local and regional radiological protection organisations.

#### The current system of Radiological Protection as set out in publication 103

ICRP Publication 103, which in 2007 laid down the currently applied System and fundamental principles of radiological protection, supports SDG 3 (Health and Well-being). By providing a System that allows society and individuals to benefit from the advantages of using ionising radiation and, at the same time, ensures the protection of patients, workers and members of the public, and the environment, the Recommendations contribute to the accessibility of safe, high-quality healthcare services, and to reduce the risk associated with exposure from air, water and soil pollution and contamination (Table 1).

As already noted, the System that has been developed by ICRP is based on science including ethics, and experience. It applies to all sexes, all genders, all ages, all ethnicities, and all countries. Thus, the System – by its very nature – contributes also to SDGs 5 (Gender Equality) and 10 (Reduced Inequalities).

ICRP Publication 103 also lays the foundation for environmental protection, which, in turn, also contributes to SDG 3 and other SDGs. Before, in the predecessor of Publication 103, ICRP Publication 60, the view was that “*the standards of environmental control needed to protect man to the degree currently thought desirable will ensure that other species are not put at risk*” (ICRP 1991). Later in ICRP Publication 91 it was noted, however, that “*there are no internationally agreed criteria or policies that explicitly address protection of the environment from ionising radiation*” (ICRP 2003). The decision to develop a framework for the assessment of radiation effects in non-human species was, however, not “*driven by any particular concern over environmental radiation hazards*,” because the system for protection of human beings “*has indirectly provided a fairly good level of protection of the human habitat*.” Rather, the decision was driven by the desire to design a framework for the protection of the environment that is “*harmonised with its proposed approach for the protection of human beings*.” Furthermore, ICRP Publication 91 highlights *“the need to demonstrate that the principles of radiological protection are consistent with a recognition that it is essential to consider the interdependence of humans and the environment to achieve**** sustainable development****”* (ICRP 2003, para 94). Consequently, in ICRP Publication 103 the aim was set to prevent or reduce “*the frequency of deleterious radiation effects to a level where they would have a negligible impact on the maintenance of biological diversity*,* the conservation of species*,* or the health and status of natural habitats*,* communities and ecosystems”* (ICRP 2007).

Subsequently, various methodological developments have been made by ICRP, and today, various tools are available such as the Reference Animals and Plants (RAPs) (ICRP 2008) or the Derived Consideration Reference Levels (DCRLs) (ICRP 2014a) that allow radiological protection of non-human biota in a consistent way. This affects, for example, water quality, and therefore supports SDG 6 (Clean Water and Sanitation), which calls for, among other things, reducing the quantity of pollutants released into water and restoring contaminated ecosystems. It also supports SDG 14 (Life Below Water) and SDG 15 (Life on Land): radiological protection is indeed crucial in preventing and mitigating the impact of radiation on ecosystems, marine life, and terrestrial environments, aligning with the goals to conserve and sustainably use these resources. The System has evolved since ICRP Publication 103 (ICRP 2007) to allow for greater consideration of the protection of the environment. However, the System continues to evolve to account for the latest scientific findings and to consider how concepts such as ecosystems services could be used - for example ICRP has established two Task Groups to address the protection of the environment: one on “Considering the Environment when Applying the System of Radiological Protection” and one on “Ecosystem Services in Environmental Radiological Protection” (www.icrp.org).

Thanks to their near global acceptance, the ICRP’s recommendations also contribute to SDG 16 (Peace, Justice, and Strong Institutions): strong regulatory frameworks and institutions for radiological protection contribute to the peaceful and fair management of radiation-related activities, preventing misuse and promoting safety. In addition, reinforcing the involvement of all stakeholders in the application of the optimisation principle, ICRP recommendations contribute, among others, to embark the societal and ethical concerns in the decision-making process (ICRP 2007, para 224).

By its very nature, the System supports SDG 17 (Partnerships for the Goals). Indeed, ICRP Publication 103 lays the foundations for national and international standards in radiological protection all over the world, fostering international cooperation and best practice. Having the same System in place globally builds trust and confidence in the recommendations, transposed guidelines, and established practices, and makes it equal for all. It also facilitates exchange and discussion on all aspects of radiological protection thanks to a common language on radiological protection, and it supports the consistent development of safe instruments and up-to-date protection methods. Perhaps even more important is the fact that ICRP’s position, through its authority and reputation that it enjoys thanks to its independence, makes it a respected and influential entity, which can naturally draw users of ionising radiation into support of sustainable development.

In general, ionising radiation is also used in many analytical methods that are useful, for example, to assess and prevent soil degradation and water pollution, to increase food and animal feed safety, to support diagnostic and prevention of non-oncological diseases, veterinary applications, public safety and terrorism prevention, mining and prospecting. The System as formulated in ICRP Publication 103 (ICRP 2007) supports all these and many other applications, thus contributing in many ways to the UN SDGs.

#### More recent ICRP publications

Typically, ICRP publications are prepared under one of its four existing committees, or (less frequently) under the Main Commission. Of the four ICRP committees, the two most naturally inclined to support the SDGs are Committee 3 (medicine) and Committee 4 (application of the System). The other two, Committee 1 (effects of radiation) and Committee 2 (dosimetry), essentially produce and consolidate biomedical and physical knowledge and support the SDGs indirectly. However, it is possible to identify a link between the SDGs and publications of all committees. In the remainder of this text, we carry out this exercise for ICRP documents published since the publication of ICRP Publication 103 (ICRP 2007).

#### Mechanisms of radiation action

ICRP Publications 131 (stem cells) (ICRP 2015d), 150 (cancer risk and plutonium) (ICRP 2021d) and 152 (detriment) (ICRP 2022b) are part of the theoretical basis of our understanding of the biological mechanisms of some radiation-related health effects and describe the way in which health risk is taken into account in the System. As with many ICRP publications, they contribute to the dissemination of knowledge and training for all those interested in the effects of ionising radiation. In this way, the publications may be regarded as supporting SDG 4 (Quality Education). ICRP publications on radiation-related effects complement reports of the United Nations Scientific Committee on the Effects of Atomic Radiation (UNSCEAR) (which has the mandate to undertake scientific evaluations of sources of ionising radiation and of the associated exposures, effects, and risks to human health and to the environment) and other international and national organisations. Taken together, they inform the System and, in that way, form the indispensable bases for any contribution of ICRP and the System to sustainability and the SDGs.

#### Dosimetry

While the above-mentioned radiation-related effects form a prerequisite to quantify radiation action and translate this into radiological protection guidelines – radiation doses form the practical level of protection. For this reason, a large amount of data has been generated and synthesised by ICRP to ensure accurate and consistent approaches to the assessment of radiation exposure. One example here is the dose coefficients in ICRP Publications 128 (for intake of radiopharmaceuticals by patients) (ICRP 2015a), 130, 134, 137, 141, 151, and 154 (for intake of workers and members of the public) (ICRP 2015c, 2016c, 2017c, 2019b, 2022a, 2024b), 144 (for external exposure) (ICRP 2020b), and 136 (for non-human biota exposure) (ICRP 2017b). Calculation of such dose coefficients required development of suitable phantoms of the human body or of RAPs. These phantoms are explained in ICRP Publications 143, 145 and 156 (phantoms) (ICRP 2020a, c, 2024d), while ICRP Publications 133 and 155 describe how they are used for internal dosimetry (specific absorbed fractions) (ICRP 2016b, 2024c). Most of these publications can for example be linked to SDG 8 (Decent Work and Economic Growth), in particular its sub-goal 8.1, which calls for “*protect[ing] labor rights and promot[ing] safe and secure working environments for all workers*”.

ICRP Publication 147 (dose quantities) clarifies the meaning of certain dosimetric quantities and, above all, proposes an approximate indicator of possible risk associated with ionising radiation, particularly as a function of age and sex (ICRP 2021a). This information is a first step towards stratifying risk. Seen from this angle, this publication supports SDG 5 (Gender Equality) and SDG 3 (Good Health and Well-Being).

ICRP Publication 148 (radiation weighting factors for animals and plants) improves the way in which the sensitivities of non-human biota to ionising radiation are considered (ICRP 2021b). This makes it possible to protect them more efficiently, and thus supports SDG 14 (Life Below Water) and SDG 15 (Life on Land). Other ICRP Publications dealing with the protection of the environment are listed in Table 1.

#### Medical applications of ionising radiation

Many recent ICRP publications deal with the use of ionising radiation in health care settings. ICRP Publications 129 (cone beam CT) (ICRP 2015b), 135 (diagnostic reference levels) (ICRP 2017a), 139 (interventional procedures) (ICRP 2018b), 140 (radiopharmaceuticals used in therapy) (ICRP 2019a), 149 (brachytherapy) (ICRP 2021c) and 153 (veterinary practice) (ICRP 2022c). All have the common aim of better protection of people (e.g., patients, workers, carers, and the public) in specific areas of healthcare. In this sense, they support SDG 3 (Good Health and Well-Being). The procedural ethical values of sustainability and solidarity are specifically discussed in an upcoming ICRP publication on ethics in radiological protection for patients in diagnosis and treatment in relation to overuse of resources, and education in health outcomes analysis (ICRP 2024e).

#### Exposures to natural sources of ionising radiation and protection of the environment

Finally, quite a few recent publications cover situations which go beyond the purely radiological or healthcare spheres. This is the case of ICRP Publications 126 (radon) (ICRP 2014b), 132 (aviation) (ICRP 2016a), and 142 (NORM) (ICRP 2019c) which deal with subjects such as the specific information to be given to a frequent airline flyer, how to take into account the risks of natural radioactive substances, and how radiological protection should be structured utilising a graded approach that also takes into account economic, societal and environmental factors in the development of the protection of people and the environment.

It is interesting to note that even prior to ICRP Publication 103, the idea to extend radiological protection to non-human biota has been explicitly developed in ICRP Publication 91 in 2003 with the framework for assessing the impact of ionising radiation on non-human species (ICRP 2003), and complemented by ICRP Publication 108 (ICRP 2008) through introducing the concept and use of reference animals and plants, and ICRP Publication 124 (ICRP 2014a) through dealing with protection of the environment under different exposure situations. In the same spirit, for the management of radioactive waste, it was also a key issue for achieving the protection of the future generations, as developed in ICRP Publication 122 (ICRP 2013a) for deep geological disposal. This is also emphasised in a forthcoming publication for the application of radiological protection to surface and near surface disposal of radioactive waste (ICRP 2024f). Apart from SDG 3 (Good Health and Well-Being), the topics covered in these publications can be associated with the SDGs directly linked to the quality of the environment (SDG 6 (Clean Water and Sanitation); SDG 14 (Life Below Water); SDG 15 (Life on Land)).

#### Ethical considerations

ICRP Publication 138 (ethical foundations of the System) (ICRP 2018a) and an upcoming ICRP publication on ethics in radiological protection for patients in diagnosis and treatment (ICRP 2024e) cover ideas included in many of the SDGs. Indeed, the values developed in these documents such as beneficence/non-maleficence, prudence, justice, and dignity are sufficiently wide-ranging to be associated either directly or indirectly, with most of the SDGs. This truth comes from the unconditional respect to which all human beings are entitled, to the need of considering the well-being of present and future generations in decisions taken now, including notably distributive, intergenerational justice as well as environmental justice. In addition, ICRP Publication 138 (ICRP 2018a) opens the reflection on the search for reasonableness in the application of the System, calling for a holistic approach and the introduction of trade off to cope with the application of the optimisation principle. In this respect, it is interesting to mention the representative attributes listed in ICRP Publication 101b to be considered in the optimisation process to select the best protective option (ICRP 2006). These attributes include the characteristics of the exposed population, the characteristics of the exposure, social considerations and values (including sustainability and equity among others), environmental considerations, non-radiation hazards, etc.

More recently, sustainable development was addressed in post-accident situations in ICRP Publication 146 (ICRP 2020d). For this purpose, the due attention is devoted to recovering sustainable and suitable living and working conditions in a reasonable timeframe for people living on affected areas (link to SDG11 (Sustainable Cities and Communities)).

Table 1 lists examples, without being exhaustive, of how recent ICRP publications are linked to SDGs.

### Recent changes in ICRP governance in relation to SDGs

In recent years, ICRP operation and management have followed the developments and trends relating to several aspects of the SDGs. This is also a part of the organisations’ governance, being a charity organisation and following the Charity Commission for England and Wales Diversity and Inclusion Strategy (Charity Commission 2019) and the UK Equity Act 2010 (UK 2010). ICRP operates nowadays in a much more open, transparent, and collaborative way than before, often by making use of modern communication technologies and reaching out to many parts of the world.

Other examples include the development of the Code of Ethics (ICRP 2024 h), the fact that the ICRP rules ensure a turnover in the Main Commission and all Committees of at least 25% each term, and the use of open calls to recruit new members before a new term begins or when members with additional expertise are needed (for the Main Commission, Committees and Task Groups).

ICRP continuously strives to improve diversity in its membership. The number of female members among Commission and Committees has increased gradually since the foundation of ICRP in 1928 and the recent data confirms the growth: from 21% for the term of 2013–2017, to 25% for the period 2017–2021 to 27% under the current term membership 2021–2025. It should be mentioned that taking Task Groups into account, the female members’ ratio is over 37%. ICRP membership also saw an increase in numbers of countries represented, by more than 20% from 2016 to 2022, indicating an increase in diversity of geographic origin.

Public consultation during and at the end of the drafting process of any ICRP publication supports a broad stakeholder involvement, allowing for an open and transparent way of developing the System. Dissemination of those publications was greatly facilitated recently through the “Free the Annals” campaign, which was successful thanks to generous donations of individuals, organisations and governments, and which offers broad access at no cost to ICRP publications subject to a two-year embargo period for the most recent publications. This approach offers easy access to most ICRP publications to everybody including those living in less privileged regions of the world or to lesser financed organisations.

The ICRP Mentorship Programme, initiated in 2019, has included up to date 76 mentees, of which 65 are currently still in the programme. The programme encourages university students and early-career professionals and scientists to join and contribute to ICRP task groups under the guidance of senior task group members. The geographic distribution of Mentees affiliation countries demonstrates increase in educational interest among the Asian and African continents.

ICRP task groups as well as all the other ICRP bodies work in an open and transparent way, which is supported by the regular organisation of virtual events where essential elements of the System can be discussed. The ICRP has held biennial symposia to increase participation in its programs and to gain deeper understanding of international radiological protection policy and practice. This is supported by formal relations maintained by ICRP with other organisations with an interest in radiological protection through specific agreements, or by granting Special Liaison status to organisations whose work is relevant to ICRP’s mandate. Organisations currently in formal relations with ICRP are listed at the ICRP website (https://www.icrp.org/icrp_group.asp?id=80).

All these actions can be linked with SDG 4 (Quality Education), SDG 5 (Gender Equality), SDG 10 (Reduced Inequalities), SDG 16 (Peace, Justice, and Strong Institutions) and SDG 17 (Partnership for the Goals).

These shifts in the ICRP structure and governance provide benefits to the radiological protection community as a whole. The year 2023 marked the midpoint in the UN SDGs, and a recent report lays out the ‘What’ and the ‘How’ to achieve success and the associated significant costs to do it (Nature 2023; UN 2023). The scientific community, including ICRP, can contribute to one crucial aspect where the report notes how we do science must change to be more multidisciplinary, equitable, inclusive, openly shared, and widely trusted, and ‘socially robust’ to be responsive to social context/needs.

## Towards the next general recommendations

To encourage the radiological protection community to strengthen their efforts and contribute to the SDGs, the issue of sustainability should be considered and addressed in the next ICRP general recommendations as the SDGs align with the fundamental mission of the Commission. As outlined above, sustainability is already an important element in the System, and ICRP has for some years instigated changes in the way it operates in line with the SDGs. Motivated by the momentum of the current UN SDG campaign, the ICRP Main Commission decided to place more emphasis on sustainable development in the updated 2024–2028 strategic priorities.

The three strategic priorities identified for the 2024–2028 term are (ICRP 2024a):

1. Keep the System of Radiological Protection fit for purpose;
2. Strengthen engagement with professionals, policy makers and the public;
3. Ensure ICRP continues to operate as a well-governed and forward-looking organisation.

### Keep the system of Radiological Protection fit for purpose

Recently, ICRP has embarked on the process to review and – as needed – revise the current System (Clement et al. 2021). This process was considered timely as the last revision took place quite some time ago and culminated in ICRP Publication 103 in 2007 (ICRP 2007). This means that the current System is based on work that had been initiated almost 25 years ago. Since then, advancement of scientific evidence, evolution of societal values, and some 15 years of experience in the application of ICRP’s most recent general recommendations may require the System to be revised (Laurier et al. 2021; Rühm et al. 2022, 2023b). In the context of the present paper, sustainability is one of the changing societal values, because it has experienced a continuously growing global awareness, particularly during the last 25 years. Consequently, it is timely to initiate discussions with ICRP’s stakeholders in relation to inclusion of sustainable development into the System.

Further integrating sustainable development into the System should enhance ICRP’s aim that efforts to protect humans and nature from the adverse effects of radiation do not compromise human health, socio-economic development, and environmental protection. Initial ideas on topics that could be relevant in this respect are given below.

### Strengthen engagement with professionals, policy makers, and the public

ICRP considers the review and revision of the System as a joint endeavour of all interested in radiological protection. For this, international relationships are key and collaboration with international organisations is essential as well as interacting with various stakeholders including civil society representatives and regional radiological protection networks. Consequently, ICRP considers many organisations as stakeholders including, for example, UNSCEAR with its secretariat serviced by the United Nations Environmental Programme (UNEP), the International Atomic Energy Agency (IAEA), the Organisation for Economic Co-operation and Development (OECD), WHO, the International Labor Organisation (ILO), the International Radiation Protection Association (IRPA), etc., and organises regular meetings to exchange views and ideas. In the spirit of SDG17 (Partnership for Sustainable Development) one way forward is to use existing relationships and intensify discussions on the link between radiological protection and sustainable development on such occasions. We envisage that those discussions could include, for example (Mayall 2022):

- Properly valuing and accounting for costs/detriments (harm) and benefits (good) of the use of ionising radiation over space, time and generations combined with a better understanding of what constitutes good and harm including the concept of well-being linked to effective communication/understanding, citizen participation and mapping to the SDGs;
- Reviewing the application of the principles of the System (Justification, Optimisation, and Application of dose limits) and – if necessary – adapt them in a way that supports sustainable development;
- Analysing whether the application of the optimisation principle would benefit from further clarification to reinforce its purpose to optimise overall protection and foster an all-hazard approach;
- Improving broad participation in how to link sustainable development with the development of the System and its application, taking greater account of the need for better informed, integrated, inclusive, and holistic risk assessment and management in society; this means further addressing ethical considerations for the application of the System in all exposure situations;
- Integrating and learning from other experiences, practices, and disciplines, one example being the many experiences during the COVID pandemic, to broaden optimisation and justification practices including economic, psychological and social impacts, environmental issues and ecosystems services;
- Amending the primary aim of radiological protection to recognise explicitly the contribution of the System to enabling sustainable development.

SDG 13 (Climate Action) should have a role in the application of the radiological protection principles, especially justification and optimisation. The System could contribute to this goal through the optimisation principle. In the current general recommendations, optimisation means that “the likelihood of incurring exposure, the number of people exposed, and the magnitude of their individual doses should all be kept **a**s **l**ow **a**s **r**easonably **a**chievable, taking into account economic and societal factors” (ICRP 2007). In ICRP Publication 146, this was already broadened to some extent, and “environmental factors” were added (ICRP 2020d). Climate action involves both mitigation (reducing greenhouse gas concentrations in the atmosphere) and adaptation and resilience to climate change. A more holistic approach than often adopted could allow/offer the carbon footprint to be considered in radiological protection principles (Picano, 2023). By way of example, recycling and reuse of lightly contaminated material is one way to save valuable resources, when proven that it would not contribute to any significant increase in the exposure of the public, workers, or the environment. Adding the dimension of sustainability to the optimisation principle could thus help to evaluate the level of protection for people and the environment with regard to the establishment of a circular economy and, in this way, support reduction of greenhouse gases and other pollutants and waste.

Many organisations have developed web-based greenhouse gas equivalencies calculators that may be used to estimate the impact of industry, and in other assessments, for example, in medical applications and in economic appraisals (EPA 2024; UK DESNZ 2020, 2023). Another approach for the medical imaging professions could be to introduce the carbon impact information into the medical radiological protection community with a checklist such as that suggested by Picano et al. (2023). Interestingly, in their checklist some of the topics (“5. *Like radiation*,* carbon cost should always be justified*“; “*6. Like radiation*,* carbon cost should always be optimized*;” “*7. Like radiation*,* the responsibility for inappropriate carbon costs should be that of both the prescriber and the practitioner*”) draw some parallels between radiation exposures and carbon emissions indicating that both scenarios share some commonalities.

The ICRP looks forward to discussing the further integration of sustainability into the System with international partners and other stakeholders. This will support the current process of reviewing and revising the System.

### Ensure ICRP continues to operate as a well-governed and forward-looking organisation

ICRP policy and practices should include operational aspects such as travel, ethical funding/investment, ethical research, procurement, equality, diversity, inclusion, openness, participation, accountability, success criteria, etc. Although the primary mission of ICRP - to assure an appropriate level of protection against the detrimental effects of ionising radiation exposure without unduly limiting the benefits associated with the use of radiation - remains highest priority, operational aspects of ICRP’s work should also consider the SDGs. In this respect relevant ISO standards may provide some guidance (ISO 2018).

Issues already considered include preferring digital meetings to face-to-face meetings whenever deemed reasonable, to reduce carbon footprint, to optimise the allocation of available resources and to enable participation of an increased diversity of experts, particularly in the context of budgetary constraints. Another action is to continue and strengthen efforts towards transparency, stakeholder involvement (in particular during the process of review and possibly revision the System), interaction with and involvement of younger generations, and enhancement of the inclusion of less privileged regions currently under-represented in the ICRP membership.

## Conclusions

It was Lauriston Taylor, the first president of the US National Council on Radiation Protection and Measurements from 1964 to 1977, who coined the phrase that “*radiation protection is a problem of philosophy*,* morality*,* and the utmost wisdom*.“ In this spirit Merril Eisenbud, a well-known radiation scientist and one of the leading scientists in radiation research during the second half of the last century, held the 7th Lauriston Taylor lecture in 1983 entitled “The human environment – past, present and future”. In the lecture, he built a bridge from radiation protection to global environmental protection in emphasising that an increase in atmospheric CO_2_ concentration, which had already been documented by measurements at the Mauna Loa Observatory, would lead to global warming and climate change (Eisenbud 1983). One of his conclusions was that “*No one country could solve the problem alone: international cooperation to an extent unprecedented at least up to the present time*,* would be required to develop the ameliorative programs*.” – a conclusion fully in line with the UN 2030 Agenda for Sustainable Development which is based on a “*spirit of strengthened global solidarity*,* focused in particular on the needs of the poorest and most vulnerable and with the participation of all countries*,* all stakeholders and all people.*” (UN 2015).

While a report of the World Commission on Environment and Development on “Our Common Future” was published in 1987 (WCED 1987) and the Rio Declaration of the United Nations was endorsed in 1992 (UN 1992), unfortunately, only quite some time later has this insight become common place all over the world and that an overall consensus has developed that a sustainable development is essential for the survival of ecosystems and humankind. Along these lines, ICRP as an international organisation has an ethical responsibility to contribute towards sustainable development.

Sustainability is already an implicit part of the current System. However, whether this could be more clearly articulated for example, through including sustainability as a core element of the System, remains to be addressed. Currently the UN SDGs are mentioned in many contexts and perhaps at times overused in order to gain political currency so that one may get the impression that in those cases other agendas are being pursued. Such “greenwashing” should be avoided as it has the potential to compromise the credibility of the UN SDG campaign. Therefore, the inclusion of sustainable development and the SDGs in the System through the three pillars: scientific evidence, ethics, and experience, must be carefully considered and developed, including the best way to express those. The challenges for the System associated with the implementation of the 2030 Agenda for Sustainable Development need to be identified and discussed with the international community interested in radiological protection, to properly address the issue of sustainability in the System.

## Data Availability

No datasets were generated or analysed during the current study.
